# Identification of components in scorpion and centipede traditional Chinese medicine formulations with potentially beneficial actions in asthma: network pharmacology and molecular docking

**DOI:** 10.1186/s41065-025-00490-9

**Published:** 2025-07-02

**Authors:** Yi-Ren Chen, Ya-Da Zhang, Wei Zhang, Bin-Qing Tang

**Affiliations:** 1 Department of Pneumology, Shanghai Municipal Hospital of Traditional Chinese Medicine, Shanghai University of Traditional Chinese Medicine, No.274 of Zhijiang Middle Road, Jing’an District, Shanghai, 200071 China; 2 Department of Pulmonary Disease, Shuguang Hospital, Shanghai University of Traditional Chinese Medicine, 185 Pu’an Road, Huangpu District, Shanghai, 201203 China

**Keywords:** Asthma, Centipede, Chinese medicine, Mechanism of action, Molecular docking, Network pharmacology, Scorpion

## Abstract

**Background:**

The aim of this study is to identify the principal active components of scorpion and centipede-derived traditional Chinese medicine (TCM) ingredients using network pharmacology and explore their mechanisms of action in the treatment of asthma.

**Methods:**

The chemical constituents and target information pertaining to scorpion and centipede-derived TCM components were obtained from the Traditional Chinese Medicine System Pharmacology (TCMSP) database and an herbal database. Asthma-related target genes were retrieved from the GeneCards and the Online Mendelian Inheritance in Man (OMIM) databases. The “component-target” network was constructed with the identified target genes using “Cytoscape 3.9.2” software, and the protein-protein interaction (PPI) network was generated in conjunction with the String database to further identify the core targets. The Kyoto Encyclopedia of Genes and Genomes (KEGG) pathway enrichment analysis and gene ontology (GO) functional enrichment analysis were carried out on the targets associated with scorpion and centipede-derived TCM components. Molecular docking was subsequently performed using Autodock Vina software to validate the results. Asthma mouse model was established, and mouse lung tissues were collected for histopathological examination. The levels of TP53, HSP90AA1, and IL-17 mRNA in the mouse lung tissues were evaluated.

**Results:**

A total of 11 active components met the screening conditions, including 4 centipede-derived components and 7 scorpion-derived components. The key components identified included histamine, L-histidine, stearin, cholesteryl ferulate, and cholesterol, among others. Targets with degree values ≥ 16 included TP53, HSP90AA1, HSP90AB1, steroid receptor coactivator (SRC), epidermal growth factor receptor (EGFR), estrogen receptor 1 (ESR1), mitogen-activated protein kinase 1 (MAPK1), mitogen-activated protein kinase 3 (MAPK3), and histone deacetylase 1 (HDAC1). The pathways involved comprised calcium signaling, estrogen signaling, arachidonic acid metabolism, inflammatory mediator and transient receptor potential (TRP) signaling, vascular smooth muscle contraction, thyroid hormone signaling, sphingolipid signaling, IL-17 signaling, insulin resistance, and human cytomegalovirus infection pathways. Furthermore, the mouse experiments showed that SC improved inflammatory cell infiltration and mucus secretion in mouse lung tissues and significantly suppressed the expression of TP53, HSP90AA1, and IL-17 mRNA (all *p* < 0.05).

**Conclusion:**

Scorpion and centipede-derived active components may exert therapeutic effects in asthma treatment through potential targets such as TP53, HSP90AA1, HSP90AB1, SRC, EGFR, ESR1, MAPK1, MAPK3, and HDAC1.

**Supplementary Information:**

The online version contains supplementary material available at 10.1186/s41065-025-00490-9.

## Background

Asthma is an obstructive airway disease characterized by chronic lower airway inflammation and reversible airflow limitation, with clinical manifestations including wheezing, dyspnea, chest tightness, and cough, which are typically variable and recurrent in nature. The pathogenesis of asthma involves complex interactions among genetic susceptibility, immunoregulatory imbalance, and environmental exposure [1–4]. Asthma is the second leading cause of death among chronic respiratory diseases. Studies have shown that in 2021, the global incidence of asthma was 3,340 cases per 100,000 population, with a higher incidence in males under the age of 20. Current treatments for asthma primarily include inhaled corticosteroids, long-acting β2-agonists (LABA) [5–7], leukotriene modifiers, and some biologics (such as omalizumab). Despite their important role in asthma management, these treatments are still associated with numerous side effects and adverse reactions in clinical practice [8]. 

Centipedes and scorpions feature among the insect-derived category of traditional Chinese medicinal ingredients, and these are known to have the effects of dispelling pathogenic wind to relieve convulsions, counteracting toxic substances, and eliminating the mass. They are widely used in the treatment of cerebrovascular diseases, bronchial asthma, and rheumatoid arthritis, among other conditions [9]. Spectroscopic data have revealed the presence of structurally diverse alkaloids in centipedes that exhibit anti-inflammatory and anti-renal fibrosis activities [10]. Certain compounds in scorpion venom, such as the enzyme peptide phospholipase A2 (PLA2), possess pharmacological potential for the treatment of various diseases, including wound healing, anticancer, antiviral, antibacterial, and antiangiogenic effects [11]. In our previous studies, we found that drugs derived from the scorpion-centipede pair could improve inflammation and asthma symptoms by reducing ferroptosis in lung epithelial cells in mice [12]. However, the specific mechanism of action of the scorpion and centipede-derived component combination in improving asthma remains unclear.

Network pharmacology is an emerging discipline grounded in systems biology theory, analysis of biological networks, multi-target drug molecule design, and identification of specific network nodes as targets in drug design [13]. Given the multi-component and multi-efficacy characteristics of traditional Chinese medicine (TCM), network pharmacology enables the construction of drug networks based on the structure and therapeutic properties of medicinal substances, thereby effectively predicting the active components of TCM [14]. 

Therefore, given the unclear mechanism of scorpion-centipede pair in treating asthma, this study adopts an exploratory research design to analyze the potential mechanisms of these Chinese medicinal herbs in asthma treatment and provide a basis for follow-up research. Specifically, this study aims to establish a dynamic and multi-dimensional research framework for the mechanisms of TCM compound formulas by integrating cutting-edge computational systems biology techniques. The research proceeds as follows: active components of the scorpion-centipede pair are screened using databases such as TCMSP; dynamic network models are constructed with Cytoscape 3.9.1; high-affinity binding targets are identified through molecular docking technology; and their biological activities are validated via animal models. This approach transcends the limitations of static analysis in traditional network pharmacology, offering novel insights into the treatment of asthma with TCM compound formulas.

## Materials and methods

### Collection and screening of active components of scorpion-centipede pair and targets of asthma-traditional Chinese medicine

A comprehensive search for centipede-related components was conducted using the keyword “centipede” within the Traditional Chinese Medicine System Pharmacology (TCMSP) database (https://old.tcmsp-e.com/tcmsp.php). Active components were then screened based on pharmacokinetic characteristics related to their absorption, distribution, metabolism, and excretion (ADME) parameters. The screening criteria included an oral bioavailability (OB) ≥ 30% and a drug likeness (DL) score ≥ 0.18 [15]. Similarly, a whole component search of scorpion-related components was done using “scorpion” as the keyword in the herb database (http://herb.ac.cn). In addition, other compounds proposed in the Whole Scorpion Centipede Remedy that may have therapeutic properties but did not meet the above criteria were included in the comprehensive analysis based on the support of available literature [16, 17]. The pubchem database (https://pubchem.ncbi.nlm.nih.gov) was then utilized to obtain the active ingredients and their SMILES numbers (Simplified Molecular Input Line Entry System), and then the SMILES numbers were imported into the SwissTargetPrediction (http://www.swisstargetprediction.ch) to predict its effective target.

Asthma-related target genes were identified by entering the keyword “asthma” in the GeneCards (https://www.genecards.org/) and the Online Mendelian Inheritance in Man (OMIM) (https://www.omim.org/) databases. The results were then deduplicated and standardized using the Uniprot database to ensure accurate retrieval of asthma-related target genes.

### Construction of the component-target network of the scorpion and centipede-derived drug pairs

The active components and corresponding targets of the scorpion and centipede-derived drug pairs were imported into the Cytoscape software (Cytoscape 3.9.1) for visualization and analysis of the component-target network. In this network, the size of the node degree represents the number of connections a node has with other nodes in the network. The higher the node degree, the more influential the role played within the network. The important active components within the scorpion and centipede-derived drug pairs were identified based on the importance of the nodes, as determined by their node degree.

### Screening of common targets between scorpion and centipede-derived drug pairs and asthma, and construction of the protein-protein interaction (PPI) network

Using Venny software (https://bioinfogp.cnb.csic.es/tools/venny/), the intersecting targets between the active compounds of scorpion and centipede-derived TCM components and asthma-related targets were identified. which can be used as potential key targets for the treatment of diseases with TCM compound ingredients. The Wayne diagram visualizes the common association of these genes between drugs and diseases. This analysis will help further understand the mechanism of action and potential therapeutic targets of centipede and scorpion-derived drug pairs in the treatment of bronchial asthma.

The TCM components-disease intersecting targets were input into the Search Tool for the Retrieval of Interacting Genes/Proteins (STRING) database (https://www.string-db.org/) to construct the interspecies protein-protein interaction (PPI) network. The screening criteria set were as follows: target species was human (*Homo sapiens*), an interaction score of 0.900 (highest confidence), including hidden free nodes, and other parameters set as their default settings. Subsequently, the PPI network diagram was generated.

Next, the data files were directly imported into the Cytoscape 3.9.2 interface for data visualization and analysis. Assessing the connectivity values and combined score values between the targets reveals the interaction among the targets. Higher connectivity and combined score values indicate stronger interrelations between targets. This analysis allows for the identification of key targets and the construction of a target-pathway network diagram for understanding the relationship between centipede and scorpion-derived TCM components and asthma.

### Network analyses of gene ontology (GO) enrichment and Kyoto Encyclopedia of Genes and Genomes (KEGG) pathway enrichment

The common genes between the centipede and scorpion-derived drug pairs and bronchial asthma were subjected to Kyoto Encyclopedia of Genes and Genomes (KEGG) pathway enrichment analysis and Gene Ontology (GO) functional enrichment analysis using the functional annotation bioinformatics analysis software Bioconductor (http://www.bioconductor.org/). Pathways with a significance level of *P* < 0.001 were selected, and the top 10 pathways with the highest number of enriched genes were chosen to create an advanced bubble plot.

The GO analysis includes three areas, providing annotations on the role of target proteins in drug therapy for diseases in gene function from three aspects: biological process (BP), cellular component (CC), and molecular function (MF). KEGG analysis focuses on pathway analysis and aims to elucidate the primary signaling pathways involved in drug therapy for diseases.

### Molecular docking

To validate the reliability of the predicted targets, molecular docking was performed to confirm the accuracy of the target predictions for the active components derived from the centipede-scorpion combination. The structure with the lowest binding energy was identified as the optimal configuration. The active component with the highest connectivity value was selected for molecular docking with key target genes. Protein crystal structure was obtained from the AlphaFold database (https://alphafold.ebi.ac.uk/). A docking small molecule library was established by searching for TCMs in the TCMSP database (https://old.tcmsp-e.com/tcmsp.php). Pymol software (PyMOL 2.5.0) and Autodock software (Autodock Vina 1.2.3) were used for protein processing, which was performed using Autodock Vina. Open Babel 3.1.1 was used for format conversion of small-molecule libraries, standardization of protonation states, and molecular charge assignment to ensure that molecular structures were adapted to subsequent docking procedures. Autodock 1.2.3 was applied for preprocessing of receptors/ligands before molecular docking (generating PDBQT files) and setting of docking parameters to predict the binding modes and binding free energies of small molecules with target proteins. A binding energy (affinity) of less than − 5 kcal mol-1 indicates good binding activity, and the smaller the value, the more stable the binding between the small molecule of the drug and the target protein receptor. The relevant methods and parameter settings were referenced from published experimental literature [18]. 

### Establishment of animal models of asthma and drug administration

BALB/c mice (6–8 weeks, 20 ± 2 g) were purchased from Jiangsu GemPharmatech Co., Ltd. The mice were housed in a specific pathogen-free environment with a 12-hour light/dark cycle, maintained at 24 ℃±2 ℃, 50–70% humidity, and provided with food and water ad libitum. Ten mice per group were placed in 2 cages, with mice from the same group distributed across different cage rack positions. Cage replacement and bedding cleaning were performed twice weekly. Cage positions were rotated clockwise daily. This experiment was approved by the Animal Experiment Ethics Committee of the Shanghai Municipal Hospital of Traditional Chinese Medicine, Shanghai University of Traditional Chinese Medicine. Mice were grouped using a complete randomization method as follows:

All 30 mice were uniformly numbered M01–M30. Random numbers were generated using the RAND function in Microsoft Excel (example formula: =RAND()). After sorting the random numbers in ascending order, mice numbered 1–10 were assigned to the blank group, 11–20 to the control group, and 21–30 to the Chinese medicine group. Grouping was performed by an independent researcher. Cages were labeled only with animal numbers (e.g., M01). Neither the experimental operators nor data analysts were aware of the group corresponding to each number.

Blinding was implemented for all animal dosing and procedures. Saline for the blank group (control group) and ovalbumin (OVA) group, as well as Chinese medicine for the scorpion and centipede-derived TCM group (SC group), were prepared and dispensed into standardized gavage tubes by independent researchers, labeled only with animal numbers (M1–M30). Experimental operators and data recorders were unaware of the group information corresponding to each number. Animal cage positions were rotated clockwise daily to avoid bias from environmental factors.

An OVA-induced asthma model was constructed based on published literature and our preliminary findings [19, 20]. We used AL(OH)3 as an adjuvant to induce and enhance asthma airway inflammatory response. Thirty mice were randomized into three groups, with 10 mice in each group: control group, OVA group, SC group. Mice were modeled after one week of acclimatization. On days 1, 7, and 14, 0.1 ml of normal saline containing OVA (2.5 mg/kg) and aluminum hydroxide (10 mg/kg) were intraperitoneally injected to establish the asthma model in the mice.

Starting on day 15, the mice were exposed to 2% OVA nebulization solution in a closed container and treated with nebulizer therapy for 21 days for 40 min per day. Mice in the control group were administered intraperitoneal injections of normal saline on days 1, 7, and 14, and were placed in a closed container filled with saline nebulization solution on day 15.

From day 15 to day 36, mice in the control and asthma groups were injected with 10 ml/kg of saline by gavage 1 h prior to each nebulization. mice in the SC group received SC solution 0.625 g/kg, both administered at 09:00 daily prior to mouse nebulization.

**Fig. 1:**
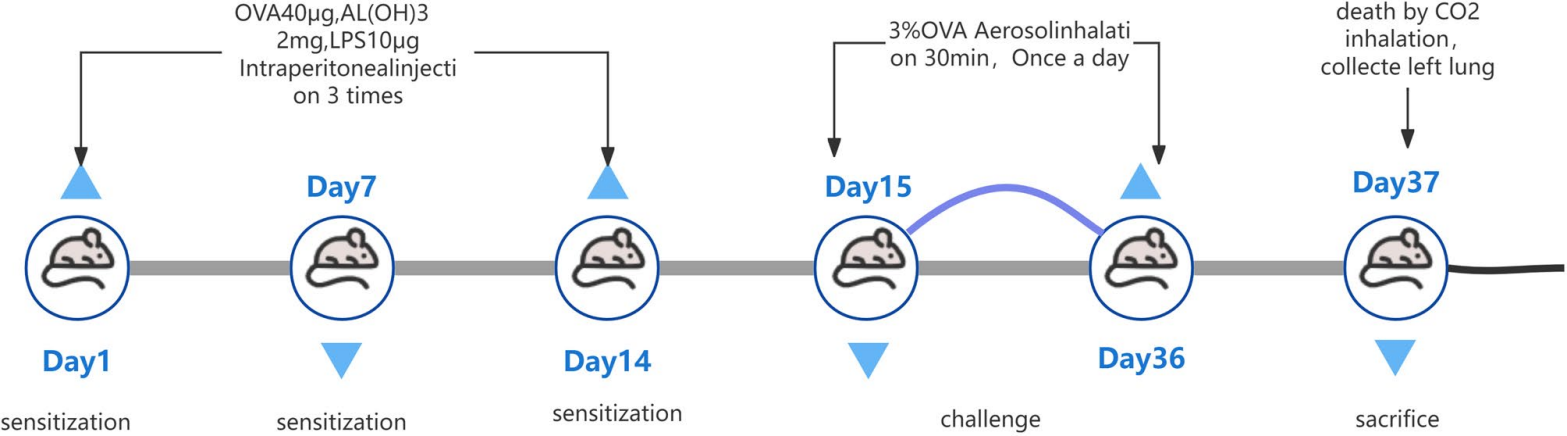
Flowchart of the animal modeling process

Twenty-four hours after the last nebulization, blood samples were collected from the mice via the orbital sinus. Serum was isolated one hour later and stored at -80 ℃. Subsequently, the mice were euthanized by carbon dioxide inhalation. The left lung of the mice was harvested and partially fixed, and the remaining tissue was kept in a -80 ℃ freezer for further experiments. A flowchart of the animal modeling process is given in Fig. 1. A total of 30 male mice aged 6 weeks (body weight 20–22 g) were included in the experiment. They were divided into a blank group, an OVA group, and a Chinese medicine group by complete randomization, with an initial allocation of 10 mice per group. No animals died or were excluded during the entire experiment, and finally, 10 mice in each group were included in the data analysis.

### Hematoxylin-eosin (HE) staining

The right lung upper lobe tissue of each group of mice was selected for HE staining. Formalin-fixed lung tissues were fixed in 10% buffered formaldehyde (Shanghai Biotechnology Co., Ltd., Shanghai, China), dehydrated, paraffin-embedded, nuclei were counterstained with hematoxylin (Shanghai Biotechnology Co., Ltd., Shanghai, China), and cytoplasm was stained with eosin. Finally, sections were dehydrated and dehydrated in gradient ethanol, and sections were sealed with neutral balsam. The pathological changes of lung tissues were observed with a light microscope (LEICA DM500, Germany).

### Quantitative reverse transcription polymerase chain reaction (qRT-PCR)

Total RNA was extracted from the lung tissue, and its concentration was determined using a NanoDrop ND-1000 spectrophotometer. Reverse transcription was performed using the Maxima First-Strand cDNA Synthesis Kit (Vazyme) for RT-PCR. Gene expression was quantified using the Taq Pro Universal SYBR qPCR Master Mix real-time fluorescence quantification PCR Kit (Vazyme) on an ABI Q6 real-time fluorescence quantification PCR machine. GAPDH was used as the internal reference gene. The relative mRNA expression levels of heat shock protein 90AA1 (HSP90AA1), tumor protein p53 (TP53), IL-17, and GAPDH were calculated using the 2 − ΔΔCt method. The sequences of all gene primers are listed in Supplementary Table 1.

### Statistical methods

In this study, GraphPad Prism 9.0 statistical software was used for data analysis and processing. Experimental results were presented as mean ± standard deviation (x ± s). All continuous variables were evaluated for normality using the Shapiro-Wilk test in GraphPad Prism. Data conforming to a normal distribution (*p* > 0.05) were analyzed by one-way analysis of variance (ANOVA). For non-normally distributed data, the Kruskal-Wallis test was applied. Homogeneity of variance was verified using the Brown-Forsythe test.

## Results

### Prediction of component targets of scorpio-centipede herb pair and potential targets of bronchial asthma

A total of four centipede-derived components were identified from the TCMSP database based on the defined ADME screening criteria, while seven scorpion-derived components were included from the herb database. After eliminating duplicates, the centipede and scorpion-derived drug pairs consisted of 11 components and 795 associated targets.

A search yielded 9,290 potential targets from the GeneCards database and 36 targets from the OMIM database. After deduplication and standardization, 9,312 asthma-related targets were identified.

### “Drug-component-target” network of the scorpion and centipede-derived drug pairs and asthma

**Fig. 2:**
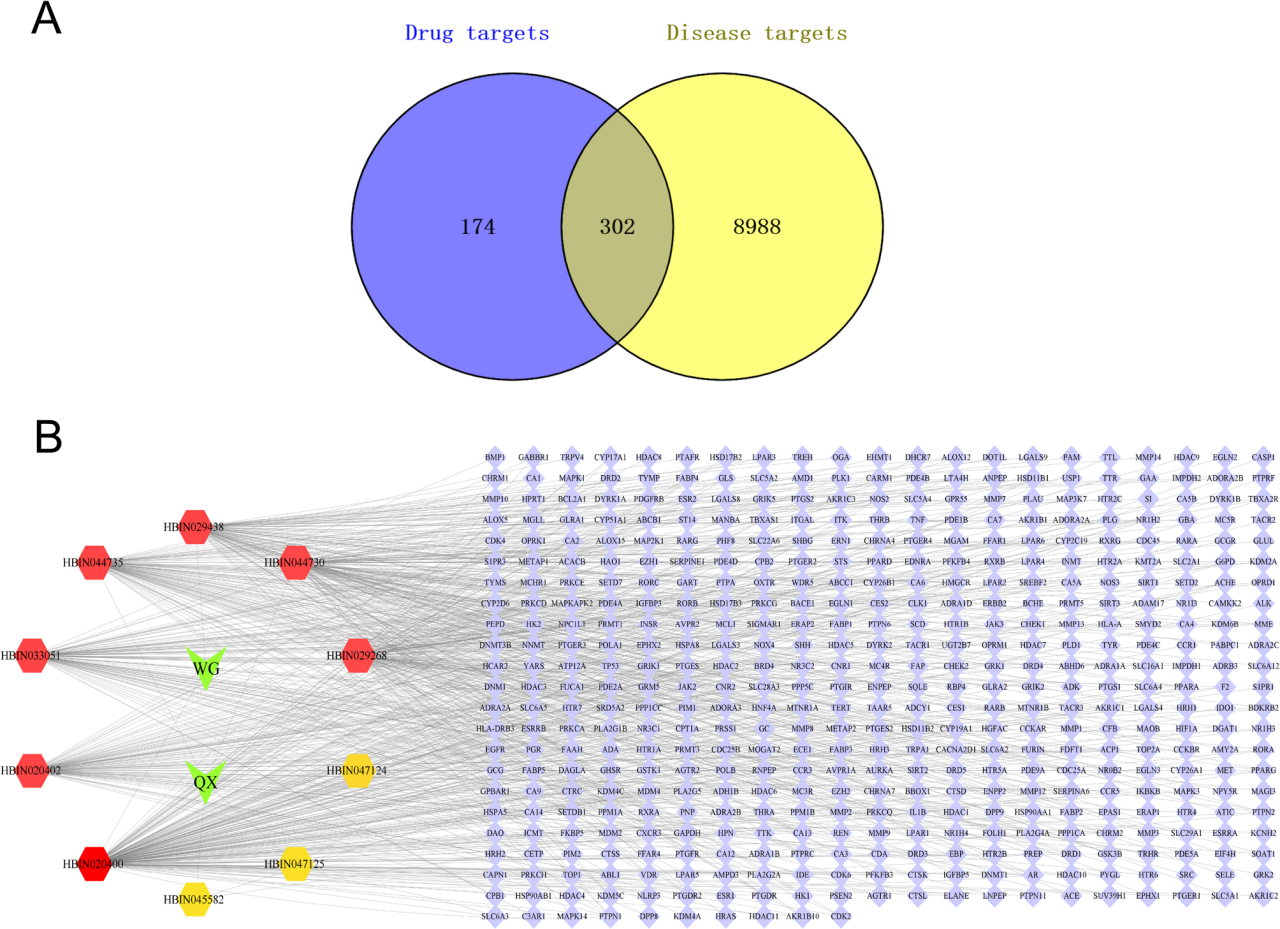
**A**: Venn diagram of the common targets of the scorpion and centipede-derived TCM components and asthma; **B**: Scorpion and centipede-derived active component-target network diagram

The drug targets derived from centipede and scorpion components as well as the asthma-related targets were imported into Venny software to identify the common targets, which were visualized using a Venn diagram (Fig. 2A). The common targets were then uploaded to the STRING database to assess interactions between them, and the resulting data were further processed and visualized using Cytoscape 3.9.0 software (Fig. 2B).

The active components and potential targets of centipede and scorpion-derived drug pairs were input into Cytoscape 3.9.0 to construct the drug-component-target network. This network comprised 58 nodes and 472 edges, with each node representing the active TCM components within the centipede and scorpion-derived drug pairs and the potential targets for the treatment of bronchial asthma, while the edges indicated interactions between these nodes.

**Fig. 3:**
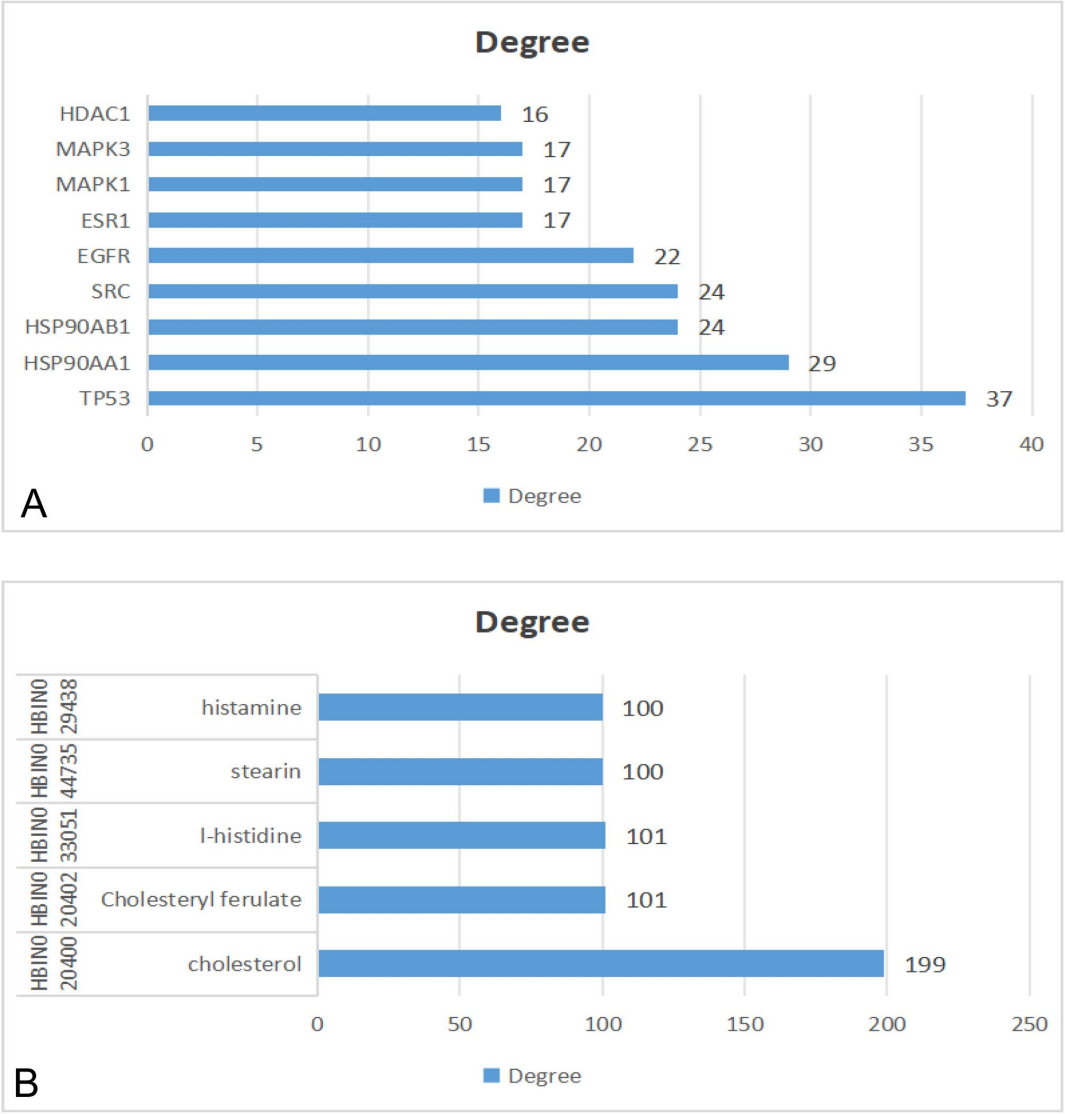
**A**: Core targets of the scorpion and centipede-derived TCM components; **B**: Key active components of the scorpion and centipede-derived ingredients

The top five active components by degree values were histamine; L-histidine; stearin; cholesteryl ferulate; and cholesterol, with values of 199, 101, 101, 100, and 100, respectively (Fig. 3). The key targets identified with degree values ≥ 16 included TP53, HSP90AA1, heat shock protein 90AB1 (HSP90AB1), Steroid receptor coactivator, SRC, epidermal growth factor receptor (EGFR), estrogen receptor 1 (ESR1), mitogen-activated protein kinase 1 (MAPK1), mitogen-activated protein kinase 3 (MAPK3), and histone deacetylase 1 (HDAC1). These components and targets are considered significant within the network.

### PPI network construction

**Fig. 4:**
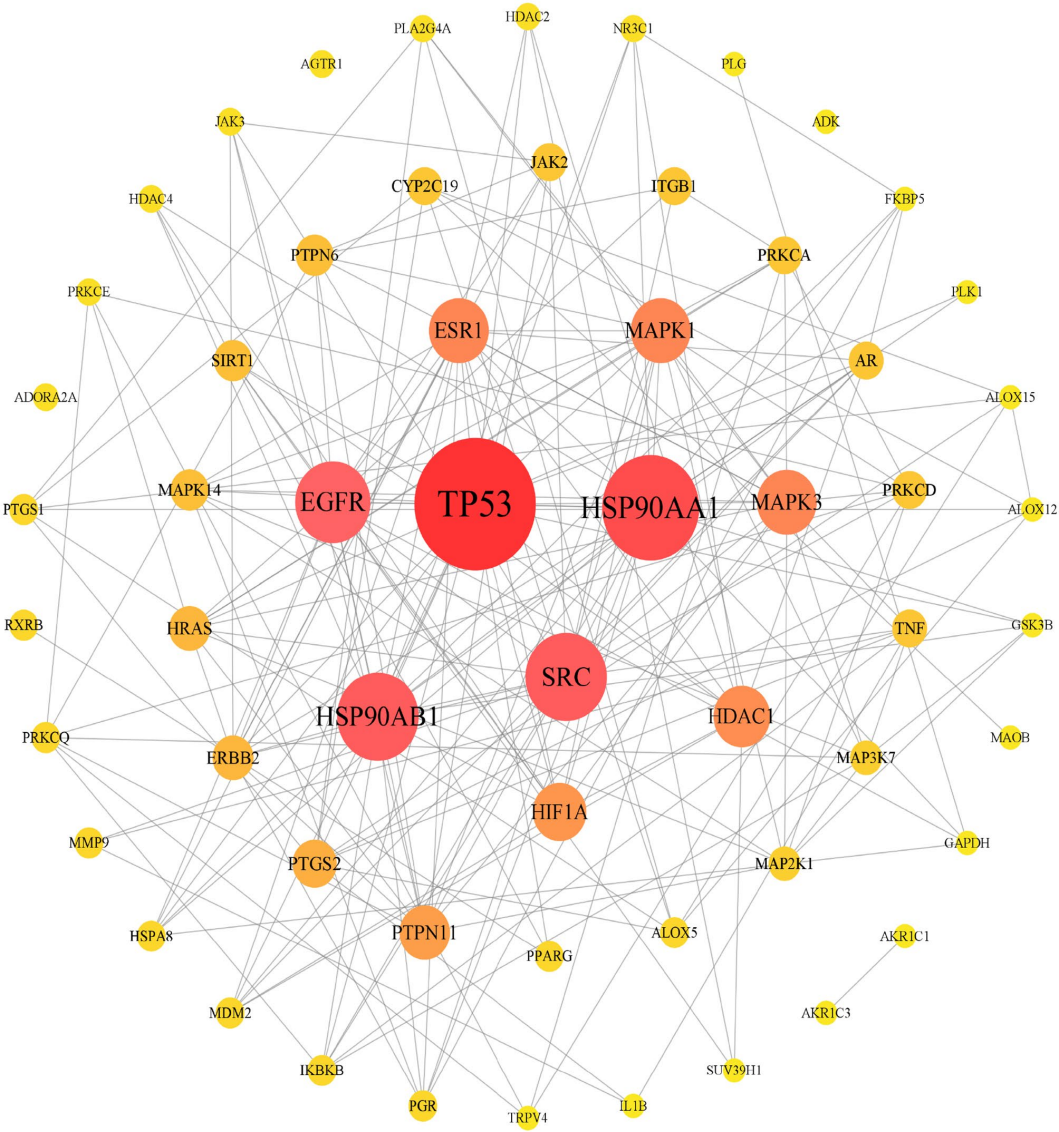
PPI network diagram of target-protein interactions of the scorpion and centipede-derived components

A total of 302 target proteins of the centipede and scorpion-derived drug pairs potentially associated with bronchial asthma were imported into the STRING database to analyze their interactions. These interactions were then visualized using Cytoscape 3.9.0 software to obtain the PPI network (Fig. 4). The network comprised 58 nodes and 472 edges. In this visualization, the larger the node, the greater its value, indicating a closer the relationship and greater importance within the network.

### GO enrichment analysis and KEGG pathway enrichment analysis

**Fig. 5:**
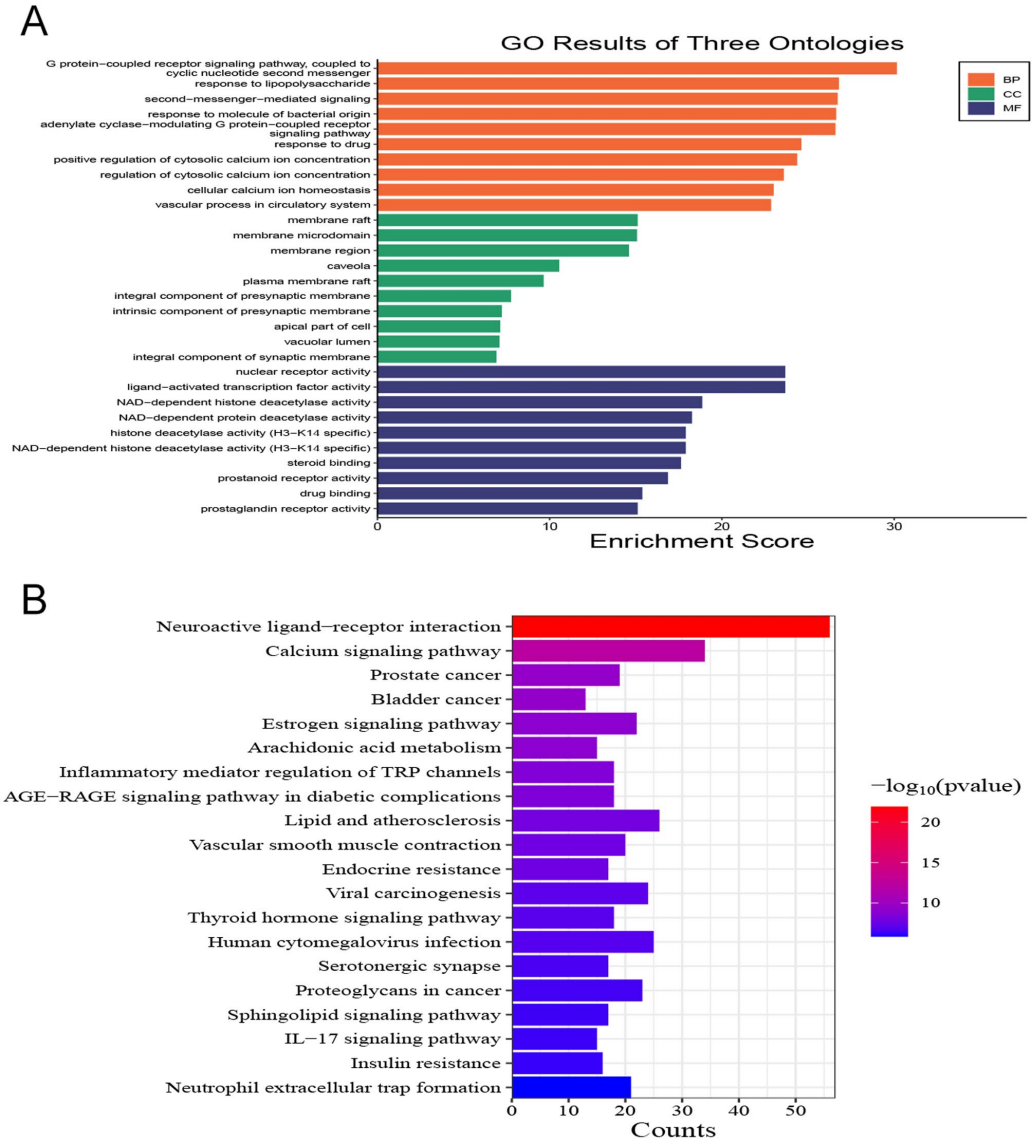
**A**: GO function enrichment analysis; **B**: KEGG pathway enrichment analysis

The GO functional enrichment analysis (Fig. 5A) revealed the following top 10 biological processes with the highest number of enriched genes: G protein-coupled receptor signaling pathway, lipopolysaccharide-mediated response, bacterial molecule-mediated response, second messenger-mediated signaling, adenylyl cyclase regulation of G protein-coupled receptor signaling pathway, positive regulation of cytosolic calcium concentration, cellular calcium ion homeostasis, nuclear receptor activity, ligand-activated transcription factor activity, and vascular processes in the circulatory system. These findings suggest that the pathogenesis of asthma involves numerous biological processes, and the scorpion and centipede-derived drug pairs may exert therapeutic effects by modulating these processes.

The KEGG pathway enrichment analysis (Fig. 5B) showed that the targets of scorpion and centipede-derived compounds in improving asthma were predominantly closely linked to several key pathways: the calcium signaling pathway, estrogen signaling pathway, arachidonic acid (AA) metabolism pathway, inflammatory mediator and transient receptor potential (TRP) signaling pathway, vascular smooth muscle contraction, thyroid hormone signaling pathway, sphingolipid signaling pathway, interleukin (IL)-17 signaling pathway, insulin resistance, and human cytomegalovirus infection.

### Molecular docking

Molecular docking was used to verify the interactions between the core proteins with high degree value identified in the PPI network and the corresponding active components of the drug. The protein crystal structures were determined by searching the AlphaFold database (https://alphafold.ebi.ac.uk/). The small molecule library for docking was established using data from the TCMSP database (https://old.tcmsp-e.com/tcmsp.php). The protein crystal structures were prepared by dehydrating and hydrogenating them using AutodockTools, followed by receptor structure preparation. The small molecule library was split using Open Babel 3.1.1 and Autodock 1.2.3 programs for further docking preparations.

**Fig. 6:**
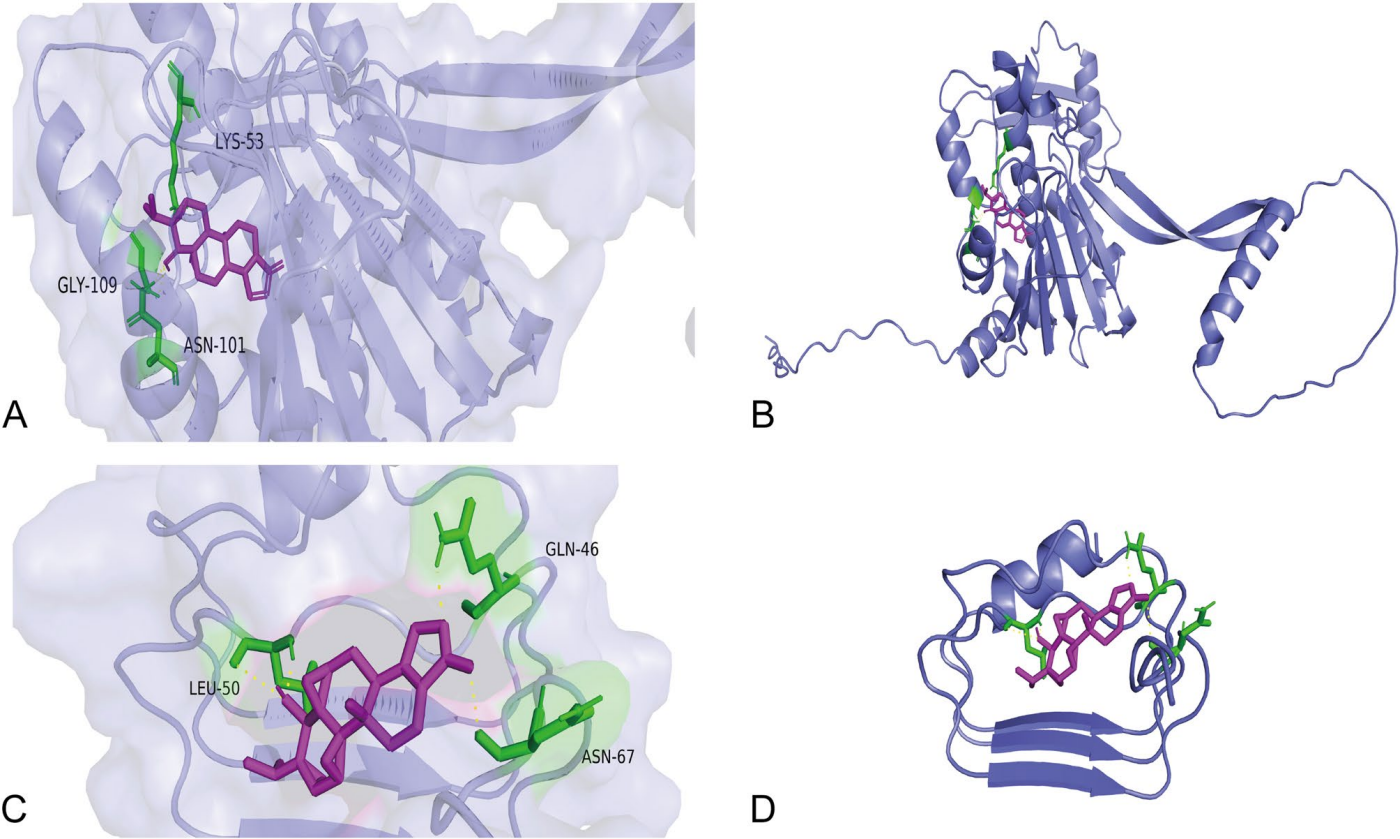
Molecular docking diagram for cholesterol. **A,B**: Molecular docking diagram of TP53 and cholesterol. **A** shows the overall results; **B** depicts the specific results. **C**, **D**: Molecular docking diagram of HSP90AA1 and cholesterol. **C** shows the overall results; **D** depicts the specific results

Docking was carried out using the Autodock program, and the results were visualized using PyMOL. The small molecule selected for docking was cholesterol, an active drug component obtained from the TCMSP database, while the macromolecules were the top two core proteins, TP53 and HSP90AA1, identified in the PPI network (Figs. 6A-D). The binding heat energy of molecular docking <-1 kcal.mol-1 indicated binding activity, and <-5 kcal.mol-1 indicated good binding activity. The PDB ID of TP53 is 8DC6 [21], and the PDB ID of HSP90AA1 is 5NJX [22]. 

According to the molecular docking results from AutodockTools 1.5.6 software, the small molecule cholesterol demonstrated good binding activity with the two macromolecular receptors with top PPI core protein degree values, TP53 and HSP90AA1, with binding heat energies of -7.6 and − 6.9, respectively. Therefore, the molecular docking results suggest a good affinity between cholesterol—an active component of the scorpion and centipede-derived drug pairs—and the core targets such as TP53 and HSP90AA1, potentially contributing to the treatment or improvement of bronchial asthma by acting on these relevant targets.

### Comparison of general conditions and pulmonary histopathological examinations among mouse groups

Mice in the Control group exhibited no signs of agitation or rapid breathing after nebulization with normal saline. Their fur was lustrous, and they were highly responsive. In contrast, mice in the OVA group showed agitation, rapid breathing, and mouth breathing after nebulization with OVA. They also had dull fur, appeared lethargic, and had reduced activity, water intake, and food consumption. Mice in the SC treatment group showed improved symptoms of rapid and mouth breathing. Their general condition improved, with more lustrous fur and increased responsiveness.

**Fig. 7:**
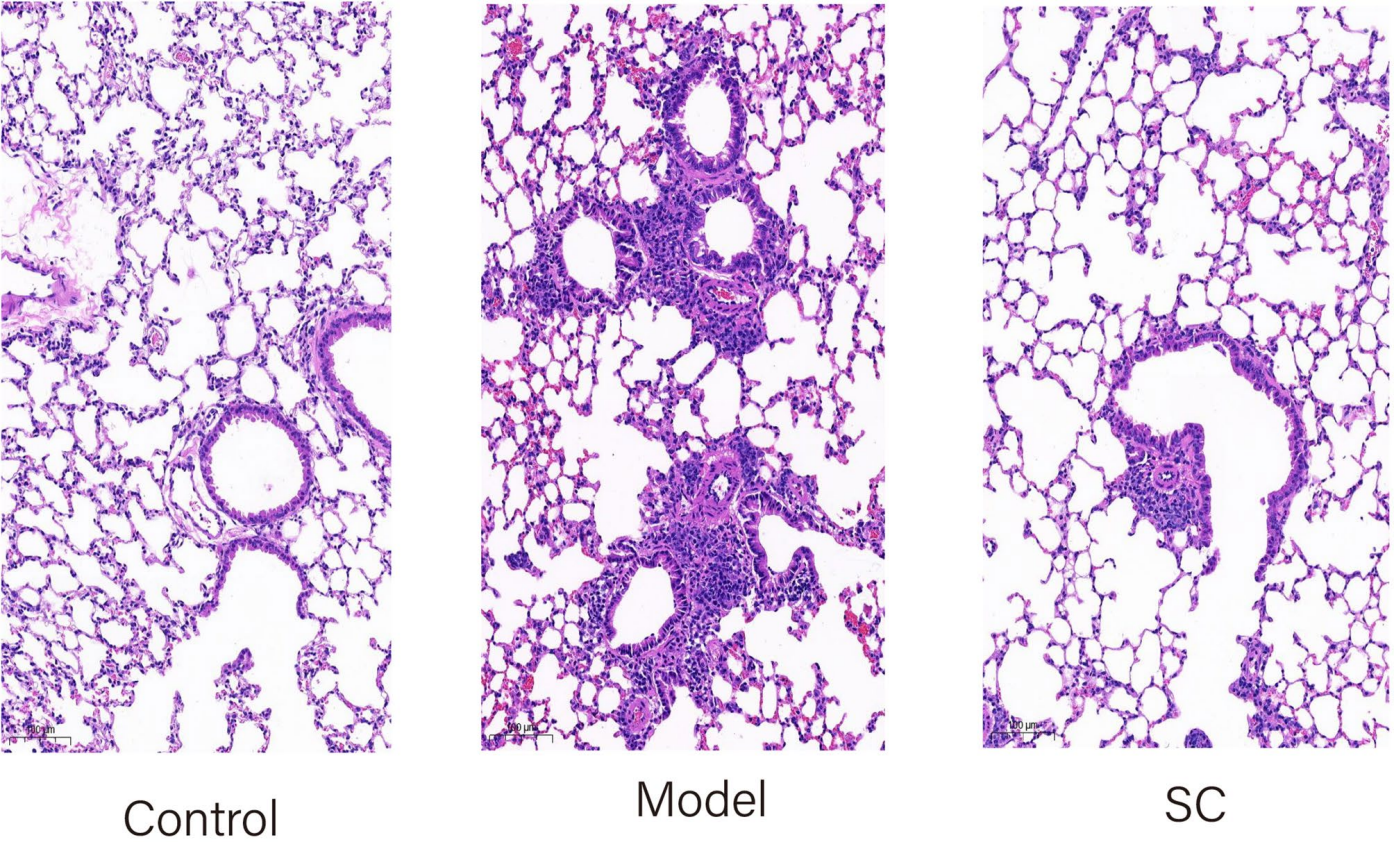
Comparison of lung histopathology among control, model, and SC-treated groups in mice. Control: Lung tissue in mice shows well-organized cellular arrangement, normal bronchial structure, absence of congestion in alveoli, and no evidence of inflammatory cell infiltration. Model: Lung tissue in mice exhibits alveolar collapse, detachment of bronchial mucosal epithelial cells, increased luminal mucus, and extensive infiltration of inflammatory cells. SC: Lung tissue in mice demonstrates a gradual restoration of alveolar morphology towards normalcy, with reduced detachment of bronchial mucosal epithelial cells, decreased luminal mucus, and diminished inflammatory cell infiltration

The results of HE showed that: the negative control group mice had well-arranged cells, normal bronchial structure, no congestion and edema in the alveoli, and no inflammatory cell infiltration; the positive control group mice showed alveolar collapse, bronchial mucosal epithelial cell detachment, increased mucus in the lumen, and a large number of inflammatory cell infiltration; the alveolar morphology of the mice in the SC treatment group was gradually restored to normal, and the epithelial cells of the bronchial mucosal were detached, luminal mucus, and inflammatory cells were reduced. mucus, and inflammatory cells decreased (See Fig. 7 for details).

### Effects of SC on the expression levels of HSP90AA1, TP53, and IL-17 mRNA in mouse lung tissues

**Fig. 8:**
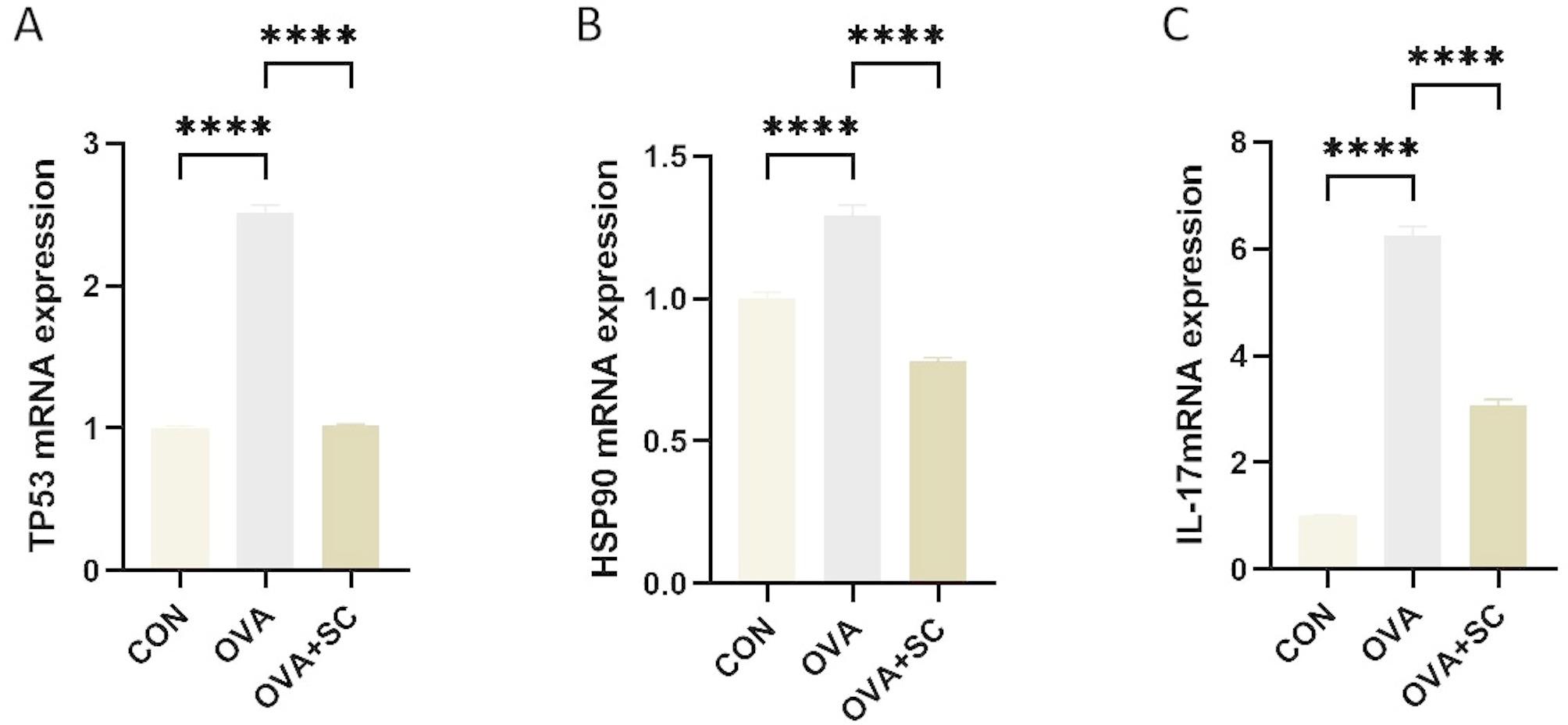
**A**: TP53 mRNA expression levels in the lung tissues in the mouse asthma model; **B**: HSAP90AA1 mRNA expression levels in the lung tissues in the mouse asthma model. **C**: IL-17 mRNA expression levels in the lung tissues in the mouse asthma model. Note: *P < 0.05, ***P < 0.001, ****P < 0.0001

Network pharmacology analysis has indicated that HSP90AA1 and TP53 are potential core targets for the regulation and control of asthma-related inflammation by the scorpion-centipede formula, while IL-17 is an important signaling pathway regulated by this formula. In the mouse asthma model, compared with the control group, the expression levels of HSP90AA1 and TP53 mRNA were significantly increased in the OVA group, whereas in the SC group, the expression levels of HSP90AA1 (Fig. 8B) and TP53 mRNA were significantly decreased (Fig. 8A). Similarly, compared with the control group, the expression level of IL-17 mRNA was significantly increased in the OVA group, but this overexpression was suppressed in the SC group (Fig. 8C).

## Discussion

The therapeutic effects of scorpion (Buthus martensi) and centipede (Scolopendra subspinipes) in traditional Chinese medicine (TCM) are characterized by their dynamic properties: “the flying ascends, the walking descends, demonstrating agility and swiftness in dispersing pathogenic factors from qi and blood stasis.” According to TCM theory, scorpion exhibits multiple pharmacological actions including calming endogenous wind to relieve convulsions, detoxifying to dissipate nodules, and unblocking collaterals to alleviate pain. Similarly, centipede possesses the properties of extinguishing wind to stop spasms, dredging meridians to relieve pain, and detoxifying to resolve masses. When used in combination, these two medicinal agents demonstrate comprehensive therapeutic effects, acting on both internal organs and external meridians, effectively resolving qi and blood stagnation throughout the body. In ancient Chinese materia medica, animal-derived medicines were collectively referred to as “chong lei yao” (insect category drugs). These zoological medicinals, being products of living organisms, are considered to possess superior efficacy in dispelling wind-phlegm, detoxifying and dissipating nodules, and resolving blood stasis to unblock meridians compared to herbal and woody medicinal substances [23]. This particular drug pair has demonstrated significant therapeutic potential in asthma treatment through the TCM mechanism of dispelling wind and dredging collaterals. The synergistic combination enables thorough searching and dredging of collaterals, effectively eliminating pathogenic factors from the collateral system while addressing the root cause of disease, thereby embodying the TCM treatment principle of addressing both symptoms and root causes simultaneously.

Asthma, characterized by airway hyperresponsiveness and airflow limitation due to chronic airway inflammation and airway remodeling, is primarily manifested as episodic cough, chest tightness, and dyspnea [24]. Despite significant advancements in diagnosis and treatment, asthma remains a serious public health challenge. Traditional Chinese medicine (TCM) serves as an effective complementary and alternative therapy. Active components of Chinese herbal medicines exert multi-level effects on asthma by regulating immune balance mechanisms or signaling pathways, demonstrating important clinical value [25]. 

TCM active components can improve asthma by modulating immune homeostasis mechanisms or influencing various signaling pathways [25, 26]. For example, catalpol, an active component extracted from *Radix astragali*, has been found to inhibit OVA-induced excessive secretion of interleukin-4 (IL-4), interleukin-5 (IL-5), eosinophil chemokines, and their receptor CC-chemokine Receptor-3 (CCR 3), thereby reducing eosinophil infiltration and alleviating airway inflammation [27]. The active compound zingerone extracted from ginger can significantly inhibit oxidative stress and inflammatory responses in asthma mouse models. It can reduce the expression of phosphorylated (p)-IκBα and p65 (nuclear), while increasing the phosphorylation expression of AMP-activated protein kinase (p-AMPK), nuclear factor erythroid-2-related factor 2 (Nrf2), and heme oxygenase-1 (HO-1), thereby alleviating oxidative damage and inflammatory responses both in vivo and in vitro [28]. In our previous study, we also found that scorpion and centipede-derived drug pairs improved asthma-induced inflammation in mice by increasing the M2 macrophage-derived exosome miR-30b-5p, reducing the expression of nucleotide-binding oligomerization domain-like receptor protein 3 (NLRP3), caspase-1, and IL-1β, and reducing mitochondrial swelling [19]. 

In our past experiments, we used high dose of salmeterol/fluticasone inhalation therapy as the basis and control group combined with tiotropium bromide inhalation therapy. And the treatment group was intervened with the combination of centipede and whole scorpion granules. The results showed that the improvement of ACT score in the treatment group was better than that of the control group, and the dosage of short-acting reliever drugs in the last week of treatment also decreased significantly compared with that in the first week, and the reduction was more obvious than that of the control group, indicating that the efficacy of the treatment was better than that of the control group in terms of symptom control [29]. It is effective in the treatment of asthma, but its specific mechanism of action is not clearly understood.

Network pharmacology, an emerging approach that integrates systems biology and pharmacology, provides a scientific framework to explain the mechanisms of action of these TCM components in the treatment of asthma. The key active components identified in the TCM active component-target network analysis included histamine, L-histidine, stearin, cholesteryl ferulate, and cholesterol, with cholesterol being particularly noteworthy. Cholesterol has been recognized as an important lipid mediator in exosomes [30], and exosomes are released from dendritic cells, eosinophils, mast cells, and bronchial epithelial cells to regulate the chronic inflammatory processes involved in asthma [31]. Cholesterol has been recognized as an important lipid mediator in exosomes, which have a unique role in anti-inflammation. An experimental analysis found significant evidence for an association between PCSK9-mediated reduction in LDL cholesterol and reduced risk of allergic asthma. PCSK9 inhibitors that reduce PCSK9 activity may be prioritized in future clinical trials investigating drugs for the prevention or treatment of allergic asthma [32]. A study revealed a linear negative correlation between serum high-density lipoprotein cholesterol (HDL-C) and blood eosinophil counts in adult asthma patients in the United States, suggesting that serum high-density lipoprotein cholesterol (HDL-C) levels may be associated with the immune status of adult asthma patients. Therefore cholesterol and the mediators involved in lipid metabolism are well worth studying in depth [33]. 

Histamine affects many cell types involved in the regulation of innate and adaptive immune responses, including antigen-presenting cells (APCs), natural killer (NK) cells, epithelial cells, T lymphocytes, and B lymphocytes. Histamine acts through H-1 receptors in the airways and can increase water secretion into the lumen, thereby modulating the viscosity of mucus released as a result of H-2 stimulation. There have been animal experiments, most notably in rhesus monkey, cat and rat trachea, and sheep bronchial tubes, which have revealed that H-2 agonists may have bronchodilatory activity. H1 antihistamines act as inverse agonists that bind to H1 receptors, thereby inhibiting histamine-induced inflammation. Extensive literature, including large-scale meta-analyses and systematic reviews, has confirmed the safety and efficacy of second-generation H1 antihistamines in the treatment of allergic rhinitis and other allergic diseases, including allergic asthma [34]. Research has shown that in patients with allergic asthma, the expression of H1R and H2R on CD 4 and CD 8 positive T cells isolated from peripheral blood is increased. Incubation of these cells with histamine could reduce the percentage of T cells staining positively for cytokines such as IL-4, IL-13, and IFNγ [35]. 

In the context of asthma treatment, some neuropeptides have been found to be localized to nerves in human airways and have a significant effect on airway smooth muscle tone, bronchial blood flow, microvascular exudation, and airway secretion. These peptides, including vasoactive intestinal peptides and related molecules like small peptide histidine-methionine. In the future, the development of drugs that interfere with or mimic the effects of neuropeptides may provide new treatments for airway diseases such as asthma [36]. Despite this potential, the specific mechanisms of action of some components such as stearin and cholesteryl ferulate remain underexplored. Further studies are required to reveal their specific mechanisms in the treatment of asthma.

This study identified TP53, HSP90AA1, HSP90AB1, SRC, EGFR, ESR1, MAPK1, MAPK3, and HDAC1 as potential core targets of the scorpion and centipede-derived TCM in treating asthma through PPI network analysis. Experimental results showed that TP53 expression was significantly elevated in an OVA-induced asthmatic mouse model, but was significantly downregulated after intervention with the Chinese herbal formula, possibly related to the inhibition of TP53 phosphorylation. HSP90 inhibition can block IL-13/IL-17-induced phenotypic transformation of airway inflammatory cells, and HSP90 signaling inactivation can enhance glucocorticoid sensitivity. Activation of the EGFR pathway promotes Th2-type immune responses and TSLP release through the MEK/ERK and p38 MAPK signaling cascades, while the MAPK family (especially the p38 subtype) plays a key regulatory role in allergen-induced airway inflammation [37–40]. Additionally, scorpion and centipede-derived TCM inhibits eosinophil inflammatory infiltration by regulating the PI3K/Akt-MAPK signaling axis [41, 42]. Collectively, this herb pair may improve asthmatic airway inflammation and immune imbalance by synergistically inhibiting TP53-mediated airway epithelial cell senescence and blocking excessive activation of the HSP90-EGFR-MAPK signaling network.

The formula significantly inhibited the expression of TP53, HSP90AA1, and IL-17 mRNA, suggesting that it alleviates airway vascular remodeling and mucosal congestion in severe asthma by intervening in IL-17 A/F release mediated by Th17 cells, blocking CXCR2 signaling-driven neutrophil recruitment, and inhibiting IL-33-induced airway hyperresponsiveness. The active components may correct lipid metabolism disorders in severe asthma by regulating 5-LOX activity, inhibiting excessive production of CysLTs, and restoring lipoxin balance. Molecular docking showed that cholesterol has high binding affinity with TP53 and HSP90AA1 (binding energy < -8.5 kcal/mol), suggesting that it may inhibit abnormal activation of the LOX pathway by stabilizing the conformation of target proteins. Components of the herb pair may also suppress IgE-mediated mast cell activation through regulating TRP ion channels (temperature/osmotic pressure-sensitive types), thereby blocking early immune responses in allergic asthma.

KEGG enrichment analysis was conducted based on the above predicted targets. The results showed that the targets of scorpion and centipede-derived components in improving asthma were mainly closely related to the IL − 17 signaling pathway, AA metabolism pathway, inflammatory mediator pathway, and the TRP signaling pathway. Experimental results in mice showed that the scorpion and scorpion and centipede-derived TCM significantly inhibited the expression of TP53, HSP90AA1, and IL-17 mRNA, suggesting that the herb pair may play an important role in asthma treatment by regulating these two core targets and the IL-17 pathway. In severe asthma, where neutrophils are dominant, T-helper 17 (Th 17) cells secrete IL-17 A, IL-17 F, IL-21, IL-22, and TNF-α which drive inflammation and airway hyperresponsiveness. This indicates that the herb pair may alleviate airway vascular remodeling and mucosal congestion in severe asthma by intervening in IL-17 A/F release mediated by Th17 cells [43]. The AA metabolic pathway plays a key role in many inflammatory diseases such as asthma and arthritis, among others. In severe asthma, the ALOX pathway is metabolically altered, leading to an overproduction of CysLTs and impaired lysin biosynthesis. Drugs that modulate LOX have also been shown to have a better rehabilitative intervention effect for asthma, and ALOX 5 or LT receptor antagonists have been developed for the treatment of asthma [44]. These findings suggest that AA metabolism pathways may be involved in the pathogenesis of severe asthma. The TRP ion channel, which can act as temperature and osmotic sensors and are involved in allergen IgE-mediated stimulation and activation of mast cells, has also been found to be potentially involved in allergic asthma [45]. 

Molecular docking results showed that cholesterol, an active component of the scorpion and centipede-derived TCM, has high affinity for the core targets TP53 and HSP90AA1 (binding energy < -8.5 kcal/mol), suggesting it may regulate key asthmatic pathways by stabilizing the conformation of target proteins. Future studies are needed to validate the interaction mechanism between cholesterol and these targets using gene-editing models and structural biology approaches, providing a basis for targeted drug development.

This study has identified potential active components and mechanisms of scorpion and centipede-derived TCM in asthma treatment through network pharmacology and molecular docking. However, the findings are based on computational predictions and limited experimental validation. Further validation of the key findings should be conducted using other models, such as the house dust mite model to enhance the generalizability of the research. The specific mechanisms of some components, such as stearin and cholesteryl ferulate, remain unclear. An increasing number of network pharmacology studies prefer to use more natural product databases while researchers directly calculate ADME parameters to screen potential active components. In this experiment, molecular docking and animal experiments were used to verify that the screening results are consistent with biological effects. Future research should focus on in-depth experimental studies to validate the predicted targets and pathways. Emerging databases such as HerbBioMap will also be further incorporated in follow-up research to enhance the breadth of predictions. At this stage, this study has limited detection of minor effects; reliance on a single animal model may restrict the generalizability of conclusions to heterogeneous human populations. These factors may affect the external validity of the results, but the core conclusions remain internally consistent under the established conditions. In future research, the sample size will be expanded, multiple animal strains will be included, and a stepped-dose design will be adopted to refine mechanistic interpretation. Additionally, clinical trials are needed to assess the therapeutic efficacy and safety of these TCM components in asthma patients. Further exploration of the interactions between these components and other biological systems may also provide a more comprehensive understanding of their therapeutic potential.

## Conclusion

In summary, in this study, the potential targets and mechanisms of scorpion and centipede-derived drug pairs in the treatment of bronchial asthma were explored using a network pharmacology approach, and the results were further validated utilizing molecular docking techniques. Our preliminary results confirmed the efficacy of these drug pairs, demonstrating their action on TP53, HSP90AA1, HSP90AB1, SRC, EGFR, ESR1, MAPK1, MAPK3, HDAC1, and other targets through their active components of histamine, L-histidine, stearin, cholesteryl ferulate, and cholesterol. The molecular docking results highlighted a strong affinity between cholesterol, an active component of the scorpion and centipede-derived drug pairs, and the core targets TP53 and HSP90AA1. These findings suggest that these components may play a crucial role in modulating the pathways associated with bronchial asthma. Our findings provide novel insights that may guide future research into the underlying mechanisms of action. However, these results are preliminary and warrant further validation through both preclinical and clinical trials to fully elucidate the mechanisms involved and confirm their therapeutic potential.

## Electronic supplementary material

Below is the link to the electronic supplementary material.

Supplementary Material 1

Supplementary Material 2

Supplementary Material 3

## Data Availability

All data generated or analysed during this study are included in this article. Further enquiries can be directed to the corresponding author.

## Abbreviations

AA: Amino Acid
APC: Antigen Presenting Cell
BALF: Bronchoalveolar Lavage Fluid
BP: Biological Process
CC: Cellular Component
CCR-3: CC-chemokine Receptor-3
COPD: Chronic obstructive pulmonary disease
COX: Cyclooxygenase
CXCR-2: C-X-C Chemokine Receptor Type 2
CYP: Cytochrome P450
EGFR: Epidermal Growth Factor Receptor
ESR1: Estrogen Receptor 1
HDAC1: Histone Deacetylase 1
HO-1: Heme Oxygenase-1
HSP90AA1: Heat shock protein 90AA1
HSP90AB1: Heat shock protein 90AB1
IFNγ: Interferonγ
IL-1β: Interleukin-1β
IL-17: Interleukin-17
LOX: Lipoxygenase
MAPK1: Mitogen-activated Protein Kinase 1
MAPK3: Mitogen-activated Protein Kinase 3
MF: Molecular Function
NK: Natural Killer Cells
NLRP3: Nucleotide-binding Oligomerization Domain-like Receptor Protein 3
NRF-2: Nuclear Respiratory Factor 2
OVA: Ovalbumin
SC: Scorpio and centipede
TH 17: T-helper 17
TNF-α: Tumor Necrosis Factor-α
TP53: Tumor protein p53
TRP: Transient Receptor Potential
TSLP: Thymic Stromal Lymphopoietin
