# Supplementary material for: Identification of components in scorpion and centipede traditional Chinese medicine formulations with potentially beneficial actions in asthma: network pharmacology and molecular docking
Source: Hereditas. 2025 Jul 2;162:120. doi: 10.1186/s41065-025-00490-9 (PMC12217821; doi:10.1186/s41065-025-00490-9)
Supplement: Supplementary file 1 — Supplementary Material 1 [file 41065_2025_490_MOESM1_ESM.docx]

Supplementary Table 1. Primers used in qRT-PCR

| Gene name | Forward prime | Reverse prime |
| --- | --- | --- |
| HSP90 | GACCCATAGGTTCACCTGTGT | AGCTCCTCACAGTTATCCATGA |
| TP53 | TAGGGGGCACCTAGCATTCA | CCATGGCAGTCATCCAGTCTT |
| IL-17 | CTGGACTCTCCACCGCAATG | GGACCAGGATCTCTTGCTGG |
| GAPDH | AAAGCCTGCCGGTGACTAAC | GCCCAATACGACCAAATCAGA |
